# MHz-Order Surface Acoustic Wave Thruster for Underwater Silent Propulsion

**DOI:** 10.3390/mi11040419

**Published:** 2020-04-16

**Authors:** Naiqing Zhang, Yue Wen, James Friend

**Affiliations:** 1 Medically Advanced Devices Laboratory, Center for Medical Devices, Department of Mechanical and Aerospace Engineering, Jacobs School of Engineering, University of California San Diego, La Jolla, CA 92093, USA; naz016@eng.ucsd.edu (N.Z.); yuw351@ucsd.edu (Y.W.); 2 Department of Surgery, School of Medicine, University of California San Diego, La Jolla, CA 92093, USA

**Keywords:** surface acoustic wave, thruster, underwater silent propulsion, acoustic streaming, propulsion force, submersibles, acoustofluidics

## Abstract

High frequency (MHz-order) surface acoustic waves (SAW) are able to generate intense fluid flow from the attenuation of acoustic radiation in viscous fluids as acoustic streaming. Though such flows are known to produce a force upon the fluid and an equivalent and opposing force upon the object producing the acoustic radiation, there is no convenient method for measuring this force. We describe a new method to accomplish this aim, noting the potential of these devices in providing essentially silent underwater propulsion by virtue of their use of the sound itself to generate fluid momentum flux. Our example employs a 40 MHz SAW device as a pendulum bob while immersed in a fluid, measuring a 1.5 mN propulsion force from an input power of 5 W power to the SAW device. Supporting details regarding the acoustic streaming profile via particle image velocimetry and an associated theoretical model are provided to aid in the determination of the propulsion force knowing the applied power and fluid characteristics. Finally, a simple model is provided to aid the selection of the acoustic device size to maximize the propulsion force per unit device area, a key figure of merit in underwater propulsion devices. Using this model, a maximum force of approximately 10 mN/cm${}^{2}$ was obtained from 1 W input power using 40 MHz SAW in water and producing a power efficiency of approximately 50%. Given the advantages of this technology in silent propulsion with such large efficiency and propulsion force per unit volume, it seems likely this method will be beneficial in propelling small autonomous submersibles.

## 1. Introduction

Efficient underwater propulsion has long been essential to the operation of autonomous underwater vehicles (AUV) [1,2]. Given the large size of most submersibles—compared to fish, bacteria, and other underwater entities—propellers are well suited for this purpose, converting easily generated rotational motion into rectilinear underwater motion. However, the long wake propellers generate cavitation and air entrainment, and the ample acoustic signal they radiate as a consequence is visually and acoustically detectable from long distances, to the extent that these signals can be used to identify the particular vehicle that produces them [3,4]. Furthermore, the associated machinery required to drive the propellers, whether from nuclear power, electrical motors, or more conventional internal combustion engines, generates detectable noise. As the size of submersibles decreases to the millimeter and smaller scales, electrical motors are all that remain to drive the propulsion mechanisms in submersibles, even though propellers are well known to suffer from poor efficiency at such small scales and furthermore produce torque steer that is difficult for a small craft to overcome [5]. Notwithstanding this issue, Li et al. [6] recently demonstrated acoustic-induced propulsion by a lead zirconate titanate (PZT) piezoelectric element used to drive a propeller as an underwater piezoelectric thruster. However, this combination combined the modest efficiency of the PZT element with the inefficient propeller to produce substantial energy loss and a low efficiency propulsion scheme. Waterjet propulsion was briefly introduced in [3], although regardless of scale, most waterjet devices still employ propellers, enclosing them within a tube instead of leaving them in the open flow with modest improvement in propulsive efficiency at small scales.

One of the key benefits of surface acoustic wave (SAW) devices is the ability to generate MHz-order acoustic waves efficiently that, because of these high frequencies, offer accelerations in excess of 10${}^{8}$ m/s${}^{2}$. Such large accelerations are not possible to generate in any other known way and are suitable for directly propelling fluids and particles in numerous micro- to nano-scale fluidics applications [7,8,9]. The acoustic radiation and fluid streaming from such devices have been the subject of many publications, from fluid manipulation, particle/cell separation, colloid and nano-object patterning, to drug delivery and more [7]. The attenuation of acoustic energy in viscous fluids along its propagation direction produces a momentum flux responsible for fluid motion: acoustic streaming. In particular, one-dimensional acoustic streaming has been investigated since at least 1948 [10], and three-dimensional bulk acoustic streaming has come to be known as Eckart streaming from that early effort [11]. In 1978, Lighthill described acoustic streaming from one-dimensional vibration of a point source in a viscous fluid [12]. Dentry et al. [13] further investigated SAW-induced acoustic streaming and improved Lighthill’s model by considering a SAW vibration area instead of the point vibration source assumption to solve the singularity problem of Sir Lighthill’s study, providing a useful tool in the analysis of microfluidics devices, but not without some work to make sense of the analysis within that.

While acoustic streaming has been developed and utilized for many applications, using it as a propulsive force has only very rarely been considered in the literature, with qualitative results more a curiosity than as a potentially beneficial mechanism. Most notably, Bourquin and Cooper [14] demonstrated the movement of a toy boat on water using a small, immersed SAW device attached to the boat. However, the underlying mechanism of SAW-induced propulsion was not examined in detail, and methods for measuring the force have not been provided in the literature. It is, however, possible to generate fluid propulsion directly from the attenuation of the ultrasound itself in beneficial ways. Hasegawa et al. [15] demonstrated an ultrasonic suction pump capable of delivering a maximum pressure of 20.6 kPa, remarkable as most acoustic pumping schemes are unable to produce more than a few tens of pascals of pressure and are therefore characterized as “flow generation devices” in racetrack flow schemes or the like instead [7,16]. Miansari and Friend [17], Zhang and Friend [18] performed fluid pumping at the nanometer scale, to produce 1 MPa pressure-driven flows, indicating a different operating mechanism that is yet to be fully explained.

Whatever the case, based on Newton’s third law, the acoustic streaming-driven propulsion of a fluid from an acoustic device likewise produces an equivalent force upon that device in the opposite direction. The measurement of this force would benefit both potential applications of such devices in micro- to nano-scale underwater propulsion and thruster devices and also aid in the characterization of the acoustic streaming generated by these devices, a phenomena that is today still only poorly understood. An aspect remarkably overlooked in the literature is the fact that because the attenuation of the sound is the mechanism for the generation of force in these devices, beyond about a meter from such a device, there is no sound to be detected. Further, even if one were very close to such a device, the frequency of the sound would be far beyond the measurement range of standard underwater probes used today on ships. Finally, there is no electromagnetic energy radiated from such devices as the acoustic wave speed is far slower than the electromagnetic field, rendering the latter “quasistatic” and therefore unable to generate strong magnetic fields. Should such a propulsion method be feasible, it would be electromagnetically and acoustically silent.

Here, we measure and model the propulsion force produced by a SAW device via acoustic wave attenuation and acoustic streaming in fluids of different viscosity. The SAW device is mounted as a simple pendulum while submerged in a fluid to quantify the propulsion force exerted upon it, using a simple force balance model that takes the orientation of the SAW device and other aspects into account. Next, a theoretical model based on acoustic radiation attenuation and force generation from the formation of an acoustic streaming jet from a finite acoustic source is provided and used to verify the experimental results and connect the measured force to the observed acoustic streaming flow field. Finally, as an example of how the method may be used, simple straight interdigital transducer (IDT) SAW devices of different sizes are compared to determine how to identify what contributes to the important figure of merit in any proposed submersible thruster: the maximum propulsion force per unit device volume.

## 2. Fabrication Methods and Materials

We fabricated interdigital transducers (IDTs) on 500 μm thick, double-side polished 128${}^{\circ }$ *Y*-rotated cut lithium niobate (LN, Jiaozuo Commercial FineWin Co., Ltd, Jiaozuo, China) for surface acoustic wave generation and propagation. A wavelength of λ=100 μm was selected for an operating frequency of ∼40 MHz (from f=v/λ) to define each IDT, comprised of twenty simple finger pairs with finger and gap widths of λ/4; frequencies less than 40 MHz for 500 μm thick LN will not produce Rayleigh SAW [7], and this is the reason for our choice of this frequency in this device. Standard UV photolithography (using AZ 1512 photoresist and AZ 300MIF developer, MicroChem, Westborough, MA, USA) was used alongside sputter deposition and lift-off processes to fabricate the 10 nm Cr / 1 μm Au IDT upon the LN substrate [7]. A dicing saw (Disco Automatic Dicing Saw 3220, Disco, Tokyo, Japan) was used to cut the entire wafer into small-sized SAW device chips. A sinusoidal electric field was applied to the IDT at resonance using a signal generator (WF1967 multifunction generator, NF Corporation, Yokohama, Japan) and amplifier (ZHL–1–2W–S+, Mini-Circuits, Brooklyn, NY, USA) to generate the SAW. The actual voltage, current, and power across the device were measured using a digital storage oscilloscope (InfiniiVision 2000 X-Series, Keysight Technologies, Santa Rosa, CA, USA).

To prepare the device for testing as a pendulum, two 50-mm segments of enameled wire (P155, 0.09-mm diameter enameled wire, Remington Industries, Johnsburg, IL, USA) were used to both deliver the electric signal and serve as pendulum strings to the device as the pendulum bobbed. Insulation was removed at both ends, and one end of each wire was soldered (2.2 % Flux Core Solder Wire, SMDSW.202, Chipquik, Niagara Falls, NY, USA) using appropriate flux (zinc chloride flux, Harris, Mason, OH, USA) to the IDT bus bar electrodes at 340 ${}^{\circ }$C.

## 3. Experimental Methods and Results

### 3.1. Quantifying the SAW Propulsion Force with a Simple Pendulum

In spite of ample research on SAW-induced acoustic streaming reported in the literature over the years, the ability of acoustic streaming to produce a reaction force upon the device that is the source of the acoustic streaming has not been investigated in detail. We present a simple force balance method by defining the SAW device as the bob in a pendulum while immersed in a working fluid. The angle of deflection of this pendulum can be used to quantify the force generated by acoustic streaming from the SAW device.

**Figure 1:**
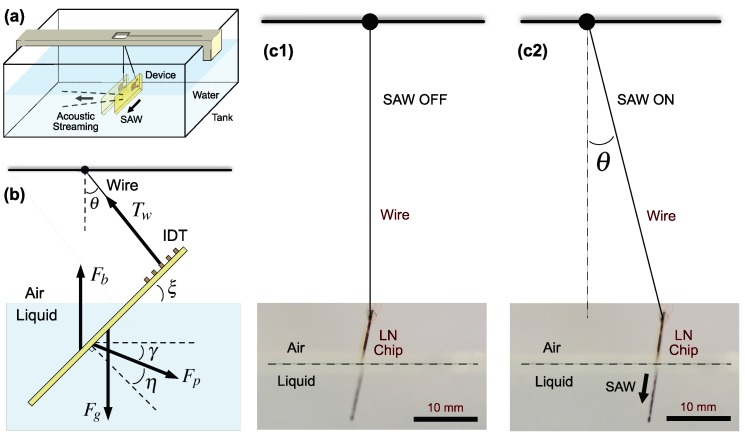
The pendulum force balancing method for measuring SAW propulsion forces via acoustic streaming, illustrated in (**a**), with the overview of a glass tank filled with the immersion fluid. A SAW device is suspended as the bob of a pendulum from a 3D-printed fixture at the top using the wires to connect the IDT of the SAW device as the pendulum arm. Taking (**b**) into account the tilted configuration of the SAW device and the Rayleigh angle η of the acoustic wave propagating from it into the fluid as it swings to an angle θ, an appropriate force balance may be formed. Images taken from the side of the setup (**c**) of a SAW device (c1) before and (c2) after activating the SAW indicates its deflection angle θ as a pendulum. The edge of the LN chip is marked in black for clarity.

A SAW device was suspended from the top of a fluid tank and immersed in the fluid (see Figure 1). Acoustic streaming from the SAW produced a force that was difficult to measure. However, by Newton’s third law, the reaction force upon the SAW device was equivalent to this force and may be calculated from the equilibrium angle θ of the pendulum,

(1) $$F_{p}=\frac{\sin(\theta )}{\cos(\gamma +\theta )}\times \left(F_{g}-F_{b}\right),$$

where $\eta =\sin^{-1}\left(c/V_{R}\right)\approx 23^{\circ }$ is the Rayleigh angle, *c* is the sound velocity in the liquid, and $V_{R}$ is the Rayleigh SAW velocity on the LN substrate. Further, ξ is the angle between the fluid surface and the substrate, $\gamma =90^{\circ }-\eta -\xi$, $F_{p}$ is the propulsion force, $F_{g}$ is the SAW device weight, and $F_{b}$ is the buoyancy force, respectively. A detailed derivation can be found in Appendix A.

The setup offers a simple and quick approach to quantify a traditionally difficult quantity to measure: the force generated by a SAW device via acoustic streaming. Measuring the pendulum angle θ and the chip orientation angle ξ from the side, with knowledge of the chip weight $F_{g}$ and buoyancy $F_{b}$, the propulsion force exerted on the chip can be simply obtained via Equation (1), where the buoyancy $F_{b}$ was considered a constant due to its negligible change over the range of chip positions defined by the angle θ in our study.

### 3.2. Making Use of the SAW Propulsion Force Pendulum Method in Modeling and Measuring Acoustic Streaming

#### 3.2.1. The Basic Theory Underpinning SAW-based Acoustic Streaming Propulsion

It is now possible to construct a theoretical model of the acoustic radiation and streaming from SAW generated upon an LN device, making use of the information provided by the pendulum force balancing method. Based on Newton’s third law, the total acoustic radiation force from the device and imposed upon the fluid is the same as the propulsion force that the fluid exerts on the device. The latter was measured in our experiments, and the former can be estimated based on the fluid flow produced in acoustic streaming.

Acoustic waves propagating through a viscous medium cause acoustic streaming within it as a nonlinear phenomenon dependent on viscous attenuation. From Lighthill’s analysis of acoustic streaming in 1978 [12], the net force per unit volume produced by acoustic streaming due to attenuation is $F=\beta \rho_{0}\bar{u^{2}}$. This results from the Reynolds stress component $\rho_{0}\bar{u^{2}}$ with an attenuation length $\beta^{-1}$, where *u* is the instantaneous velocity of the one-dimensional flow, and $\bar{u^{2}}$ is the time-average of the square of instantaneous velocity. Another useful way of writing the net force per unit volume *F* is $F=\beta c^{-1}I$, where the intensity (energy flux) *I* is a vector of magnitude $c\rho_{0}\bar{u^{2}}$ directed along the sound propagation direction [12], where *c* is the speed of sound in the fluid. This leaves the force *F* as a function of the acoustic intensity.

In a narrow beam, at a distance Xfrom an acoustic source emitting power *P*, the intensity integrated across the area of the beam has magnitude:

(2) $$I\left(X\right)=Pe^{-\beta X},$$

equal to the power remaining in the beam [12]. The force produced by attenuation of the acoustic wave per unit length is obtained by integrating the force per unit volume across the area of the beam,

(3) $$F\left(X\right)=\beta c^{-1}I\left(X\right)=\beta c^{-1}Pe^{-\beta X}.$$

The total force is the integration of this value along the entire length of the acoustic wave propagation into the fluid,

(4) $$F_{tot}=\int_{0}^{\infty }F\left(X\right)dX=\int_{0}^{\infty }\beta c^{-1}Pe^{-\beta X}dX=c^{-1}P,$$

representing a rate of momentum delivery equal to $c^{-1}$ times the rate of energy delivery. Notice that changing the rate of attenuation β does not change the total force applied, although it greatly alters its distribution (3) along the beam.

Based on this result, the total acoustic force only depends on the speed of sound, *c*, in the fluid and the input power *P*. Notably, the force is independent of the value of the fluid viscosity. Therefore, defining a dimensionless parameter $F_{tot}c/P$ may serve to indicate how well the device transforms input power into output power as a propulsive force times the speed of sound.

#### 3.2.2. Measurements of the SAW-driven Acoustic Streaming Propulsion Force

**Figure 2:**
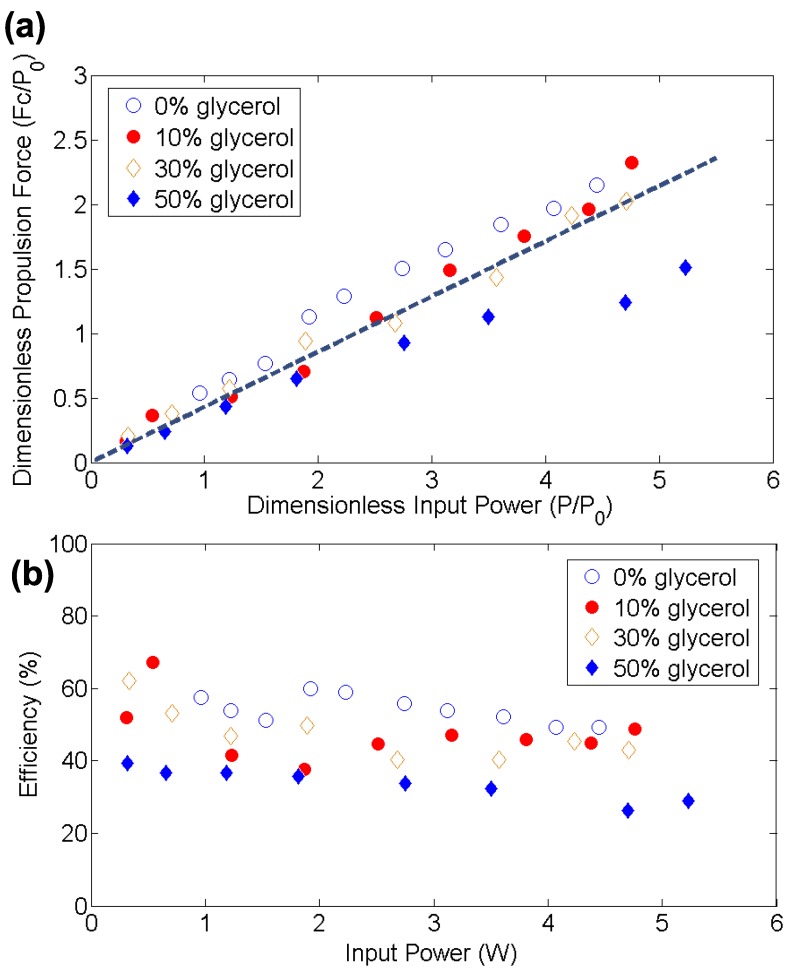
(**a**) Dimensionless propulsion force versus input power in water/glycerol solutions, at 0%, 10%, 30%, and 50% volume of glycerol with the remainder water volume, indicating a linear relationship between the dimensionless propulsion force and input power regardless of the fluid viscosity. (**b**) The electromechanical efficiency of the SAW thruster for propulsion is about 40%–60% depending on the applied input power, with greater efficiency in lower viscosity media.

Propulsion forces generated by ∼40 MHz SAW were investigated using a range of applied power and fluid viscosities. The nondimensional propulsion force $F_{tot}c/P_{0}$ is plotted in Figure 2a with respect to the nondimensionalized input power $P/P_{0}$, where $P_{0}=1$ W is a nominal reference value. The speed of sound in a water and glycerol mixture may be represented by:

(5) $$c=\sqrt{\frac{\kappa }{\rho }},$$

where κ and ρ are the bulk modulus and density of the water and glycerol mixture, respectively [19,20,21,22]. As shown in Figure 2a, the nondimensional propulsion force is linearly correlated to the nondimensional applied power. It is likewise independent of the fluid viscosity, as defined using water mixed with glycerol at the volume ratios 1:0, 0.9:0.1, 0.7:0.3, and 0.5:0.5. The quality of the linear fit shows the utility of the theory in predicting the power transmission.

As measured, the propulsion forces were smaller than expected in a 50/50 water/glycerol mix when the input power exceeded ∼4 W. This appeared to be a consequence of fluid heating due to viscous losses at these high input powers. Non-contact temperature measurement of the surface of the SAW device in contact with the fluid indicated that its temperature exceeded 60 ${}^{\circ }$C at ∼4 W input. Because LN is not hysteretic, the temperature increase was mainly due to viscous losses in the adjacent fluid. Such an increase from the lab standard of 25 ${}^{\circ }$C affects both the viscosity and speed of sound in the fluid. Notably, increasing the temperature of glycerol decreases its speed of sound, the opposite of what happens in water [23]. At high temperature, as the ratio of glycerol to water increases, the speed of sound in fluid decreases. This causes the dimensionless propulsion force to be less than the expected value at large values of the dimensionless input power, exactly as observed in Figure 2a.

As shown in Equation (4), the output propulsion power generated by the SAW thruster was predicted to be P=Fc. The electromechanical efficiency of the SAW propulsion thruster could be defined as the ratio of output power Fc to the input power, which is plotted in Figure 2b. The efficiency ranged from 40% to 60%, independent of input power, and was only weakly dependent on the fluid viscosity. Overall, SAW thruster propulsion was rather efficient compared to typical propellers, especially at smaller scales [24,25].

### 3.3. Visualization of Acoustic Streaming Responsible for the SAW Propulsion Mechanism

As the force on the SAW thruster device was produced entirely by the surrounding fluid, the visualization of the SAW-induced fluid motion near the LN chip would help elucidate the SAW propulsion mechanism. Although the acoustic radiation force that produced the propulsion force could not be visualized, the acoustic streaming that caused it could be clearly seen via particle image velocimetry.

**Figure 3:**
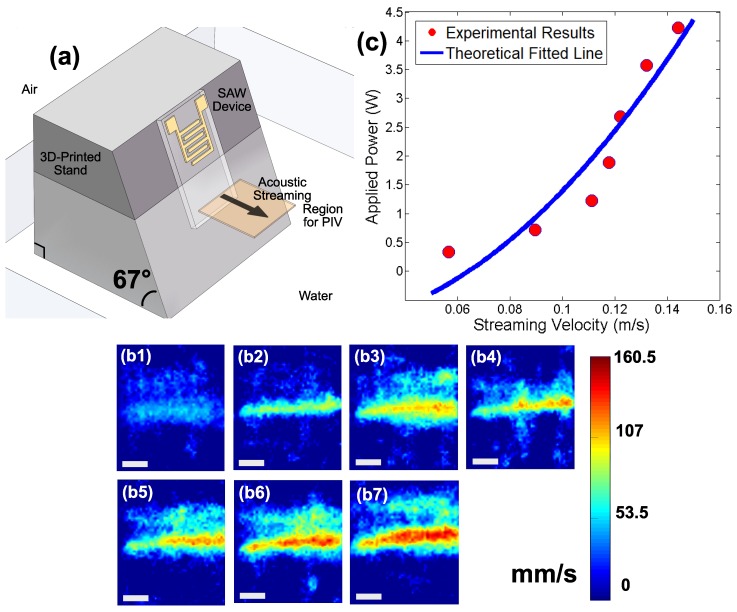
(**a**) Mounting the SAW device to visualize the acoustic streaming velocity field that it generates. (**b**) With the SAW device at the left, a top view of the PIV-derived acoustic streaming flow profile in 90% water/10% glycerol indicates that acoustic streaming increases as the applied power is increased from (b1) 330 mW, (b2) 710 mW, (b3) 1.22 W, (b4) 1.89 W, (b5) 2.68 W, (b6) 3.57 W, and (b7) 4.23 W, respectively. Scale bar: 2 mm. This produces a maximum fluid flow speed in the acoustic streaming jet as (**c**) plotted with respect to the applied power, with a fitted line based on the theoretical model of the acoustic streaming phenomena.

To reduce the complexity of visualizing the region of interest where fluid motion occurred, the SAW thruster device was mounted on a 3D-printed stand at a 23${}^{\circ }$ incline as shown in Figure 3a. The inclined plane compensated for the leaky SAW Rayleigh angle so that the acoustic wave propagating in the fluid did so horizontally. Fluorescent particles (43 μm polyethylene, Cospheric, Santa Barbara, CA, USA) were added to the fluid to track the corresponding velocity field. The particle motion was recorded by a high-speed camera (FASTCAM Mini UX100, Photron, Tokyo, Japan) and microscope (K2/CF–1, Infinity, Boulder, CO, USA) and analyzed to produce flow speed measurements (PIVlab in MATLAB, Mathworks, Natick, MA USA).

For 90% deionized water and 10% glycerol, intensity maps of PIV analysis showed the velocity field of acoustic streaming with different applied input power (see Figure 3b). Higher power produced faster acoustic streaming flow, while the width of the acoustic streaming-induced jet was similar to the IDT aperture. Conserving the fluid momentum, the change of fluid momentum in forming the acoustic streaming jet came from the output power such that:

(6) $$P\chi ∼\rho hav^{2},$$

where *P* is the applied input power, χ is the electromechanical transmission efficiency, ρ is the fluid density, *a* is both the IDT aperture and the effective acoustic streaming width, *v* is the streaming velocity, and *h* is the depth of acoustic streaming. The relationship $P∼v^{2}$ fit well with the experimental results shown in Figure 3c and was consistent with prior experimental results [12,13,26]. It indicated inertially dominant jets in which the viscous entrainment was negligible compared to the convection within the jet.

### 3.4. Using the SAW Propulsion Force Measurement Method to Improve the Propulsion Efficiency of the SAW Device

Another crucial aspect for practical underwater propulsion applications is to maximize the propulsion force for a given propulsor size. Using our simple pendulum force measurement method, the propulsion force these devices produce in water is plotted as a function of input power.

#### 3.4.1. Propulsion Force Measurement Using Three Specific SAW Device Designs

**Figure 4:**
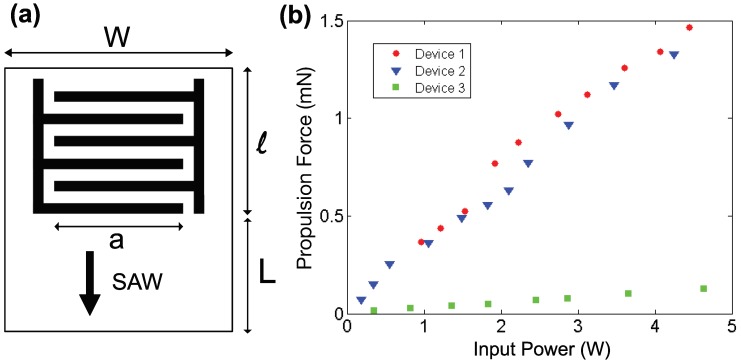
(**a**) The layout of the basic SAW device design in our trials using the pendulum force balance method. We denote the chip width, the IDT aperture, the IDT length, and the chip length for SAW propagation and attenuation in fluid as *W*, *a*, *l*, and *L*, respectively. We only consider SAW propagated towards the bottom of the chip, as the SAW propagating upward from the IDT is absorbed by an absorber mounted at the top edge of the chip. Three versions of this design produce (**b**) substantially different propulsion forces for an input power from 0 to 5 W.

To investigate the relationship between the size of the SAW device propulsion force and the propulsion force, we fabricated three different SAW device designs with sizes from ∼10 mm${}^{2}$ to ∼170 mm${}^{2}$, as depicted in Figure 4a, tabulating the SAW device width *W*, IDT aperture *a*, IDT length *l*, and SAW propagation length *L* in Table 1. Using our simple pendulum balancing method, we performed propulsion force measurements using a range of different applied powers with these devices (see Figure 4).

From the experimental results shown in Figure 4b, the propulsion forces were linearly dependent on the input power across the different SAW device designs, confirming our theoretical result in Equation (4). Comparing the results of Designs 1 and 2, the propulsion force was of the same order of magnitude, despite significant differences in the chip width *W*, IDT length *l*, and SAW propagation length *L*. The two designs, however, used the same IDT aperture *a*. With a greater SAW propagation length *L* in Design 1, SAW tended to transfer more energy to the fluid in this design and consequently generated a slightly larger propulsion force than Design 2. The propulsion forces from Designs 2 and 3 were significantly different, despite the similarity of their dimensions *W*, *l*, and *L*, because the IDT aperture *a* was quite different. In particular, Design 3, with its small ∼10 mm${}^{2}$ size, suggested it was indeed possible to fabricate a SAW thruster for microscale object propulsion.

#### 3.4.2. Model to Maximize the Propulsion Force Density: the Propulsion Force Per Unit Device Volume

The experimental results from a small selection of different SAW chip sizes and designs indicated there were significant differences in the propulsion force per unit chip volume at the same applied power. We discuss and present a calculation method here to improve the SAW device configuration and maximize the propulsion force density, in other words the propulsion force per unit device volume.

We considered a straight IDT device with dimensions as depicted in Figure 4a. The IDT aperture *a* directly affected the propulsion force: increasing it increased the force as shown in Figure 4b. Presuming the chip width *W* was chosen for a given application, the aperture should be maximized and therefore be as close to *W* as possible, but due consideration should be also given to the potentially adverse effects of having high capacitance in wide IDT designs [27] where *a* is large. Choosing to make the device smaller such that both *W* and *a* were small and the capacitance was not a problem was beneficial to increase the propulsion force per unit device volume. The IDT length *l* depended on the frequency and the number of finger pairs that formed the IDT. With a specific SAW resonance frequency in mind, the finger width and the gap between two fingers were each defined as a quarter wavelength in this elementary design. More sophisticated designs are possible, although the basic principle of defining the IDT geometry from the selected wavelength still applies. As for the number of finger pairs, one typically makes a balanced choice between the desired quality factor and the coupling performance of the substrate [7]. Taking the most common 128${}^{\circ }$ *Y*-rotated cut LN as the substrate for example, the ideal number of finger pairs was 21 with a bandwidth of 0.05. Thus, the IDT length *l* was mainly determined by the device properties and electromechanical effects. Finally, the length of the device over which the SAW was allowed to propagate, *L*, depended on the need to attenuate the SAW without reflection from the edge of the device. The energy absorbed in the attenuation of this SAW generated sound propagating in the adjacent fluid, and that led to acoustic streaming. Choosing the length *L* required slightly more effort than for *a*, *W*, or *l*.

We denote the propulsion force per aperture width as $F_{tot}$ if the SAW radiation was entirely transferred to the fluid. Based on Equation (4), the propulsion force depended only on the applied power if the sound velocity in the fluid was constant. Though we could apply a greater power to produce a larger propulsion force, we instead investigated the maximum propulsion-to-surface-area efficiency based on the SAW chip size while using the same input power. We thus assumed $F_{tot}$ to be constant as we explored the relationship between the propulsion force and the SAW propagation length *L*.

Due to the attenuation effect of the SAW propagation across the chip, the actual propulsion force per aperture width is:

(7) $$F_{p}=F_{tot}\left(1-e^{-\alpha L}\right),$$

where $1/\alpha ={\left[\left(\rho_{f}c\right)/\left(\rho_{s}v_{s}\lambda_{SAW}\right)\right]}^{-1}$ is the attenuation length for SAW on a substrate coupled with a fluid [13]. The variables $\rho_{f}$, *c*, $\rho_{s}$, $v_{s}$, $\lambda_{SAW}$ are the fluid density, sound velocity in the fluid, substrate density, SAW velocity on the substrate, and SAW wavelength, respectively. Thus, the propulsion force for a SAW device per device chip area, *A*, can be written as:

(8) $$\frac{F_{p}}{A}=\frac{F_{tot}\left(1-e^{-\alpha L}\right)a}{W(l+L)}=\frac{Fa}{W}f\left(L\right),$$

which is maximized by setting the derivative $f^{\prime }\left(L\right)=0$, producing the following for the appropriate value of *l*:

(9) $$e^{\alpha L}-\alpha L=\alpha l+1.$$

The optimal choice for *L* was calculated and is provided in Table 1 for the reader’s convenience. With a better understanding of the SAW-induced propulsion mechanism in hand, we now discuss the effects of the device size and IDT configuration on the propulsion force, aiming to provide a method for improving the propulsion force density of the device for acoustic-induced thruster designs.

## 4. Discussion

The purpose of this work is to both provide a simple method for measuring the force generated by acoustic streaming and to present a possible means for silent underwater propulsion for small to microscale submersible craft. To this end, we considered the effects of viscosity on the acoustic streaming and confirmed that indeed the force generated by the SAW device was independent of the fluid viscosity. We also considered a few simple SAW device designs to illustrate how the force balance method could help to identify what aspects of the design were most important in improving its potential use as a small thrust producing device.

The pendulum force balancing method quantifies the propulsion force exerted onto the SAW device in a simple way. Bourquin and Cooper [14] tested the SAW propulsion force at a lower frequency by measuring the drag force of a small toy vessel to which the device was attached. However, this complicated the measurement of the force by also including the effect of hull drag from the vessel. At a higher frequency of 40 MHz, we also ensured we obtained Rayleigh SAW in a similar 0.5 mm thick LN wafer, which is not possible when using frequencies lower than this. A shorter acoustic attenuation length was an added benefit in seeking a miniaturized propulsor. Producing approximately 1.5 mN with 5 W input power in a 25 mm${}^{3}$ device volume, or 60 kN/m${}^{3}$ propulsion force density, the force at first glance appeared small, but was significant considering its volume. The magnitude of the propulsion force may furthermore be easily increased by multiplexing the devices. Altogether, compared to ultrasonic thrusters using PZT [28,29], the use of SAW via LN devices appeared to provide stronger propulsion effects.

Next, a theoretical model of acoustic wave attenuation in the production of acoustic streaming and consequent fluid flow explained both the propulsion force measurement results and silent thruster performance. By offering an efficiency of 40% to 60%, the SAW thruster device appeared to possess a far higher efficiency than ultrasonic thrusters (3.9%∼33.6%) [28] and was comparable with propeller-driven thrusters (∼50%) [3,30]. With a linear relationship between the propulsion force and input power and because of the absence of hysteresis in LN, the efficiency would remain constant rather than decrease as the input power increased, as shown in Figure 2b from 0 to 5 W, attractive especially at higher power levels where other methods tend to have reduced efficiency.

Finally, a few SAW devices of different designs and configurations were considered as a cursory application of the pendulum force balance method in discerning what was important to maximize the output force, and the key combination of a large SAW aperture and a sufficiently long attenuation area beyond the IDT were found to be crucial in maximizing the device performance.

In future research work, different SAW frequencies and IDT designs could be considered using this approach in order to both seek improved thruster performance and to determine how these devices generate the acoustic streaming flows that are known to be so useful today. Dentry et al. [13] discussed, in particular, the effect of frequency on acoustic streaming, showing that the choice of frequency was crucial in the design of micro-/nano-scale devices employing acoustic streaming. For SAW-induced propulsion, the effects of frequency may also be a major aspect to optimize, particularly if different fluids and viscosities are anticipated. The broader exploration of viscous fluids may also provide additional information on the acoustic streaming phenomena. Finally, in an effort to devise practical and functional SAW-induced silent propulsion devices, integration of control and driver circuitry will be necessary in the device to facilitate its easy introduction, on par with the simplicity seen in propeller-based propulsion.

## Figures and Tables

**Table 1:** Dimensions for three different SAW devices. Units: mm.

	*W*	*a*	*l*	*L*
1	11.85	4.50	9.00	5.00
2	5.65	4.50	4.55	3.00
3	3.70	0.38	3.00	3.00

**Figure A1:**
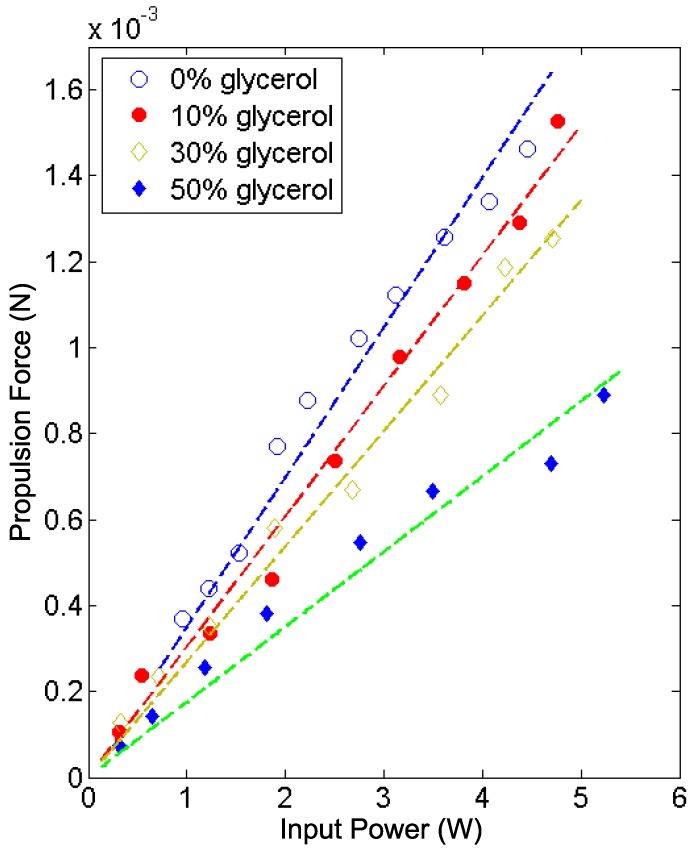
SAW propulsion force based on input power within different fluid media, indicating different propulsion force under various input power and fluid viscosity.

## Abbreviations

The following abbreviations are used in this manuscript:

SAW	Surface acoustic wave
IDT	Interdigital transducer
LN	Lithium niobate
PZT	Lead zirconate titanate

## Appendix A. Derivation of SAW Propulsion Force based on Pendulum Equilibrium

According to a force balance, the components of force in the horizontal and vertical directions are:

(A1) $$\begin{array}{c} T_{w}\cos\left(90^{\circ }-\gamma -\theta \right)+F_{b}\cos\left(90^{\circ }-\gamma \right)=F_{p}+F_{g}\cos\left(90^{\circ }-\gamma \right)\\ T_{w}\sin\left(90^{\circ }-\gamma -\theta \right)+F_{b}\sin\left(90^{\circ }-\gamma \right)=F_{g}\sin\left(90^{\circ }-\gamma \right) \end{array}$$

where $T_{w}$ is the tension from the wire, $F_{b}$ is the buoyancy force on the substrate, γ is the angle between the propulsive force and the water surface, θ is the angle of the wire relative to the vertical direction, and $F_{p}$ is the propulsion force produced by the SAW device. Combining the two Equations (A1), the propulsion force is found to be:

(A2) $$F=\frac{\sin\theta }{\cos(\gamma +\theta )}\left(mg-F_{b}\right).$$

The calculated propulsion force is plotted with different input power and fluid media in Figure A1.
